# Osteocyte specific responses to soluble and mechanical stimuli in a stem cell derived culture model

**DOI:** 10.1038/srep11049

**Published:** 2015-06-09

**Authors:** William R. Thompson, Gunes Uzer, Kaitlyn E. Brobst, Zhihui Xie, Buer Sen, Sherwin S. Yen, Maya Styner, Janet Rubin

**Affiliations:** 1 Department of Physical Therapy, School of Health and Rehabilitation Sciences, Indiana University, Indianapolis, IN 46202; 2 Department of Medicine, University of North Carolina, Chapel Hill, NC 27599.

## Abstract

Studying osteocyte behavior in culture has proven difficult because these embedded cells require spatially coordinated interactions with the matrix and surrounding cells to achieve the osteocyte phenotype. Using an easily attainable source of bone marrow mesenchymal stem cells, we generated cells with the osteocyte phenotype within two weeks. These “stem cell derived-osteocytes” (SCD-O) displayed stellate morphology and lacunocanalicular ultrastructure. Osteocytic genes Sost, Dmp1, E11, and Fgf23 were maximally expressed at 15 days and responded to PTH and 1,25(OH)_2_D_3_. Production of sclerostin mRNA and protein, within 15 days of culture makes the SCD-O model ideal for elucidating regulatory mechanisms. We found sclerostin to be regulated by mechanical factors, where low intensity vibration significantly reduced Sost expression. Additionally, this model recapitulates sclerostin production in response to osteoactive hormones, as PTH or LIV repressed secretion of sclerostin, significantly impacting Wnt-mediated Axin2 expression, via β-catenin signaling. In summary, SCD-O cells produce abundant matrix, rapidly attain the osteocyte phenotype, and secrete functional factors including sclerostin under non-immortalized conditions. This culture model enables *ex vivo* observations of osteocyte behavior while preserving an organ-like environment. Furthermore, as marrow-derived mesenchymal stem cells can be obtained from transgenic animals; our model enables study of genetic control of osteocyte behaviors.

## Introduction

A variety of stimuli converge on bone cells to regulate bone quality and density^1^. Although osteoblasts and osteoclasts are the effector cells responsible for bone matrix deposition and resorption respectively, evidence implicates osteocytes as integral orchestrators of bone remodeling through secretion of humoral signals such as RANKL^2^, sclerostin^3^ and DMP1. In addition to osteocyte regulation of local bone remodeling, osteocytes secrete factors that control renal phosphate homeostasis and bone matrix mineralization^4^. As such, osteocytes are emerging targets for pharmaceuticals aimed at controlling the release of proteins that regulate bone and phosphate metabolism^5^. However, as the osteocyte is encased in bone, it has proven difficult to study both *in vivo* and with current cell culture models.

Removing osteocytes from their spatial environment affects their phenotype and functionality. Derived from mesenchymal stem cells, some osteoblasts become encased in bone matrix within calcified tissue and attain the osteocyte phenotype. Osteocytes sends out long cellular projections through canalicular tunnels generating an interconnected network; through this lacuno-canalicular system (LCS), osteocytes receive and send regulatory signals to effector bone cells and extra-osseous tissues^6^. Osteocytes are the primary source of sclerostin (Sost)^7^, a paracrine signal that alters osteoblast differentiation; and fibroblast growth factor 23 (Fgf23)^8^, an endocrine peptide involved in phosphate metabolism^9^. Early stage osteocytes express E11 (podoplanin); a glycoprotein involved in formation of dendritic processes^10^, and other phosphate handling regulators including dentin matrix protein 1 (Dmp1) and phosphate regulating endopeptidase homolog, X-linked (Phex)^11^.

The ability of osteocyte secreted sclerostin to inhibit bone formation by blocking the Wnt/Lrp signaling axis in osteoblasts^12^ has garnered tremendous medical attention due to its potential to impact conditions of bone frailty. Clinical studies, using a sclerostin-inhibiting antibody, provide promising results for targeting bone disease^5^. Sclerostin expression is regulated by both hormonal and mechanical cues. Treatment with parathyroid hormone (PTH) suppresses Sost expression *in vivo*^3^. Similarly, mechanical loading decreases circulating sclerostin^13^, while unloading enhances its expression^14^,^15^. However, many questions remain regarding the mechanisms regulating sclerostin production and secretion, and its paracrine control of other osteocyte products. A model that rapidly produces functional Sost protein and accurately reflects *in vivo* osteocyte behavior is necessary to advance the understanding of the role of this important glycoprotein in bone physiology.

The three-dimensional environment of osteocytes is essential to their morphology and function, and their dendritic connections to the extracellular matrix are crucial for many aspects of osteocyte physiology^16^. Primary osteocytes have been used to make *ex vivo* observations; however, isolation of these cells is difficult, resulting in a heterogeneous population that provides only a short-lived phenotype in the absence of the three-dimensional matrix connections. Immortalized cell lines, such as the MLO-Y4 cells, have been widely used by our group^17^,^18^,^19^ as well as others, and have provided important insights into osteocyte biology; however, they also lack a three-dimensional environment, retain the large T-antigen, and do not produce sclerostin^20^ or FGF23^21^. These limitations raise questions as to the reliability of these models to adequately reflect the *in vivo* osteocyte phenotype. A more recently described cell line, IDG-SW3, represents a non-homogenous population progressing from early osteoblasts to late osteocytic cells. The IDG-SW3 cells only express the large T-antigen under permissive temperatures and produce abundant matrix, enabling osteocyte-like cells to develop in a more native, three-dimensional environment^21^. While this new cell line overcomes some of the limitations of previous models, changing incubation temperatures introduces an additional step in the culture process and more importantly, increases in Sost and Fgf23 expression require three weeks in culture with production peaking at five weeks, limiting rapid and reproducible experimentation.

We have developed an osteocyte model, “Stem Cell Derived-Osteocytes” (SCD-O), which faithfully recapitulates the osteocyte phenotype. These cells are generated by osteogenic differentiation of bone marrow mesenchymal stem cells (MSCs) and produce abundant osteoid, thereby mimicking the morphology and matrix interactions of osteocytes *in vivo*. Long dendrite-like processes were found projecting outward from these cells. Relative expression of osteocyte genes, including Sost, Dmp1, E11, and Fgf23 were upregulated between 10 and 15 days in culture. Expression of several of these osteocyte genes was altered by addition of hormonal and mechanical stimuli, in most cases mimicking *in vivo* responses. The development of mineralized nodules in these cultures results in dense “nodes” of osteocytic cells. While the overall culture contains a heterogeneous population of osteogenic cells, this system enables more physiologically relevant osteocytic responses by mimicking the *in vivo* environment. Additionally, the mdMSC precursors used to generate SCD-O cells can be isolated from transgenic animals, allowing the study of specific genetic influences on osteocyte physiology and bone development. The ability to produce osteocytes *ex vivo* using MSCs represents a powerful tool to study the mechanical, hormonal, and morphological features of osteocytes *ex vivo*.

## Methods

### Reagents

Fetal bovine serum (FBS) was obtained from Atlanta Biologicals (Atlanta, GA). Culture media, trypsin-EDTA, antibiotics, and phalloidin-Alexa488 were from Invitrogen (Carlsbad, CA). Ascorbic acid, and β-glycerophosphate were purchased from Sigma Aldrich (St. Louis, MO).

### Antibodies

The anti Sclerostin antibody (Ab63097) was purchased from Abcam and has been previously validated in bone tissue for Western blotting and immunostaining^22^. This Ab generates a band at approximately 25 kDa, consistent with the documented molecular weight of sclerostin and with our experimental findings. The E11 antibody (AF3244) was obtained through R&D Systems (Minneapolis, MN) and has been previously validated for Western blotting and immunostaining in calcified tissue^23^.

### mdMSC Isolation and Culture Conditions

Marrow-derived MSCs (mdMSCs) from 8–10 wk old male C57BL/6 mice were prepared as previously described^24^,^25^. Briefly, tibial and femoral marrow was collected in RPMI-1640 media (Invitrogen) with fetal bovine serum (FBS, 9%, v/v), horse serum (HS, 9%, v/v), penicillin/streptomycin (100 μg/ml) and L-glutamine (12 μM). Non-adherent cells were removed and Passage 1 cells collected after 4 weeks and re-plated in a single 175-cm^2^ flask. Passage 2 cells were plated at 2 weeks at 50 cells/cm^2^ in Iscove’s Modified Dulbecco’s Media (IMDM), and frozen in liquid nitrogen at passage 4 or 5.

SCD-O cells were generated by plating mdMSCs at 26,000 cells/cm^2^ in six-well dishes (Corning, Corning, NY). The following day, IMDM media was replaced with an osteogenic media of α-MEM, ascorbic acid (50 μg/ml) and β-glycerophosphate (10 mM), which was changed every 48 hrs.

### Electron Microscopy Imaging

mdMSC cells were plated on 60 mm Permanox dishes (Electron Microscopy Sciences, Hatfield, PA). At 15 days in osteogenic medium, SCD-O cells were fixed overnight in paraformaldehyde (4%, v/v), glutaraldehyde (2%, v/v), and ruthenium hexamine trichloride (RHT, 0.7%, w/v) in sodium cacodylate buffer (0.05 M) at 4 °C. SCD-O nodules were washed 2X with sodium cacodylate buffer (0.05 M) and dehydrated in ascending acetone (25%, 50%, 75%, and 95%) for 15 minutes each, left in ethanol overnight at 4°, followed by two ethanol washes (100%) for 15 min each. Quetol resin (EMS) was used to embed SCD-O cells according to the manufacturer’s protocol. Briefly, a quetol/n-butyl glycidyl ether (NBGE) mixture was added to SCD-O cultures in ascending ratios (1:3, 1:1, 3:1, and 100%) for 2 hours each, followed by 100% quetol overnight and an additional 1 hour incubation with 100% quetol. Resin was then allowed to polymerize at 60 °C for 48 hours.

SCD-O cells, embedded in resin blocks, were cut into ultrathin sections with a diamond knife, stained with a saturated solution of uranyl acetate in methanol, followed by Reynold’s lead citrate, and imaged with a Zeiss Libra 120 TEM at 120 kV. Images were acquired with a Gatan Ultrascan 1000 2k x 2k digital camera (Pleasanton, CA, USA).

### Western Blotting

Whole cell lysates were prepared, separated on polyacrylamide gels, transferred to polyvinylidene difluoride (PVDF) membranes and incubated with epitope specific antibodies as previously described^26^.

### Immunofluorescence

Cells were cultured in osteogenic media for 15 days, fixed with paraformaldehyde (4%, v/v) for 20 min, permeabilized with Triton X-100 (0.1%, v/v) for 5 min at RT, and donkey serum (5%, v/v) blocking buffer diluted in TBS was added for 30 min to block non-specific epitopes. Cells were washed three times for 10 min each with TBS. For actin stress fiber staining, cells were incubated with phalloidin-conjugated Alexa Fluor-488 (Invitrogen) diluted in TBS (1:100) for 30 min at RT. Cells were washed three times for 10 minutes each, covered, and sealed with mounting medium containing Dapi (Invitrogen). For antibody staining, cultures were incubated with primary Ab overnight, washed 3X with TBS, and incubated with Alexa Fluor-555-congugated secondary antibodies (Invitrogen) diluted in TBS (1:200) for 1 hr. After TBS wash, samples were sealed and mounted as above. Images were taken using a Zeiss LSM 710 confocal laser scanning microscope running ZEN 2011 software (Carl Zeiss Microscopy, Inc., Thornwood, NY), excitation 488 or 555 nm line of the argon ion laser, emission 493–630 nm, objective lens 20 × 0.95 Plan Apo.

### Real Time PCR

Total RNA was isolated using Trizol (Life Technologies, Grand Island, NY), reverse transcribed, and each gene was amplified as previously described^27^. PCR products were normalized to Gapdh or 18 S amplicons.

### Statistical Analysis

Statistical variance was expressed as the means ± SE. Statistical significance was evaluated by using either an unpaired t-test or a one-way ANOVA where appropriate (Prism GraphPad, La Jolla, CA). Multiple comparisons within ANOVAs were determined using Tukey’s multiple comparisons test. All experiments were replicated at least three times to assure reproducibility. Densitometry data, where given, were compiled from at least three separate biological replicates.

## Results

### Osteocyte gene expression occurs within 15 days

**Figure 1:**
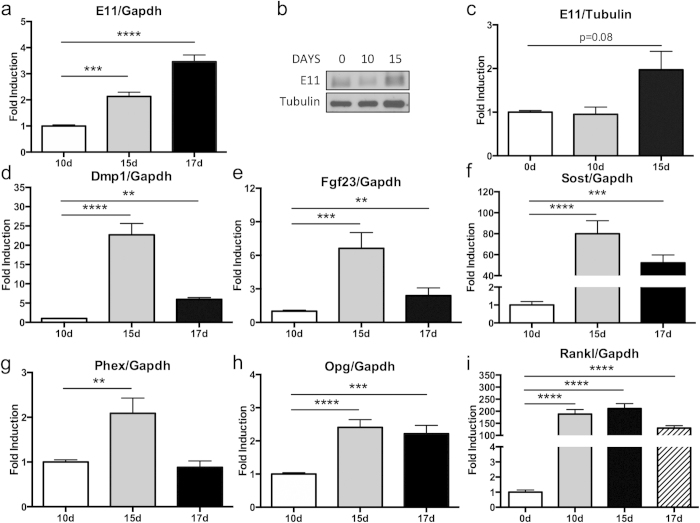
Osteogenic differentiation induces osteocyte specific gene expression. (**a**) Osteogenic differentiation for either 15 or 17 days significantly enhanced E11 mRNA expression. (**b**) Protein expression was also elevated at 15 days in culture and confirmed by densitometry (p = 0.08, compared to day 0). Blot was cropped for clarity; full-length blot is presented in supplementary figure 1. All Western blots were run under the same experimental conditions. qPCR demonstrated significantly increased expression of (**d**) Dmp1, (**e**) Fgf23, (**f**) Sost, (**g**) Phex, and (**h**) Opg at 15 or 17 days in osteogenic conditions. (**i**) Rankl expression peaked at day 10. Values were normalized to day 10 for all assays except Rankl, which was normalized to day 0. At least three replicates were completed for each assay. *p < 0.05, **p < 0.01, ***p < 0.001, ****p < 0.0001

mdMSC cells were cultured in osteogenic media for approximately 10 days before mineralized nodules containing SCD-O cells began to form. As osteocytes are defined by their relationship to the matrix, we hypothesized that osteocyte formation, and thus osteocyte marker expression, would be coincident with nodule formation. Early osteocyte markers include E11 (gp38), Dmp1, and Phex, while genes such as Sost and Fgf23 appear in mature osteocytes. By day 15, E11 mRNA expression was significantly increased by 2-fold (p < 0.001) compared to day 10; expression at day 17 was even greater with an increase of 3-fold (p < 0.0001) (Fig. 1a). Protein expression of E11 was also analyzed by Western blot, confirming enhanced E11 production at day 15 (Fig. 1b,c). Expression of Dmp1, was enhanced 23-fold in SCD-O cultures at day 15 (p < 0.0001) and to 6-fold by day 17 when normalized to day 10 (p < 0.01) (Fig. 1d). Expression of Phex mRNA, an endopeptidase that regulates matrix mineralization and renal phosphate reabsorption^4^, peaked at day 15 with an increase of 2-fold compared to day 10 (p < 0.01) (Fig. 1g).

Fgf23 and Sost are critical endocrine and paracrine signals produced by osteocytes. Fgf23 mRNA expression was significantly increased at both 15 (6.6-fold, p < 0.001) and 17 days (2-fold, p < 0.01) (Fig. 1e). Sost transcripts were measured at day 15 (80-fold, p < 0.0001) and day 17 (52-fold, p < 0.001) (Fig. 1f) and were highly elevated relative to day 10 cultures. As these osteocyte signals are essential for regulation of various physiologic responses emanating from bone, this method to generate osteocyte-dense cultures provides a robust model to study the effects of Fgf23 and Sost.

Osteocytes also produce factors that regulate osteoclast formation, including RANKL and osteoprotegerin (OPG)^2^. Real-time PCR analysis demonstrated a significant increase in Opg expression in SCD-O cells at both 15 (2.4-fold, p < 0.0001) and 17 days (2.2-fold, p < 0.001) compared to day 10 (Fig. 1h). Rankl levels significantly increased between days 0 and 10 by 188-fold (p < 0.0001), and remained elevated at day 15 and 17 (Fig. 1i). While both Opg and Rankl are known to be produced by osteocytes^2^, they are also expressed early during osteogenic differentiation.

### SCD-O cells replicate *in vivo* osteocyte morphology and ultrastructure

**Figure 2:**
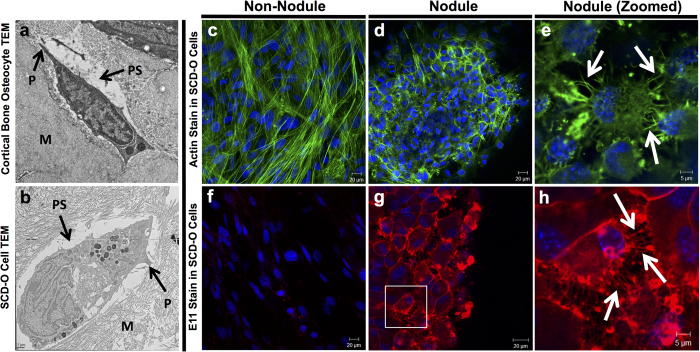
SCD-O cells replicate morphology and ultrastructure of *in vivo* osteocytes. TEM imaging of SCD-O osteocytic cells at culture day 15 (**a**) and osteocytes from murine cortical bone (**b**) showing the pericellular space (PS), surrounding matrix (M), and dendrite-like processes (P). Confocal images of SCD-O cultures (day 15), outside of mineralized nodules (**c**, **f**) or within nodules (**d**, **e**, **g**, **h**). Cells stained for actin (green) (**c**–**e**) or with an Ab to anti-E11 (**f**–**h**). Cells in nodules had small cell bodies with discrete actin stress fiber staining localized to cell processes, characteristic of osteocytes (**d**). Zoomed images highlight numerous cellular extensions on osteocytic cells (**e**, white arrows). Cells outside nodules show actin distributed throughout the cell with no cellular projections and large cell bodies (**c**). E11 staining was not observed in cells outside nodules (**f**) whereas osteocytic cells within nodules had strong E11 localization (**g**) with signal highly concentrated in osteocyte processes (**h**, white arrows).

Prior to the discovery of genetic markers specific to osteocytes, these cells were identified by their morphology and spatial relationships within bone: their long dendrite-like processes enable networked connections and the fluid-filled pericellular space enables transport of secreted factors and induces shear stress during loading^28^. The key features of osteocytes are highlighted in the transmission electron microscopy (TEM) image of an osteocyte from cortical bone (Fig. 2a). These defining features are also found in SCD-O cells. Electron microscopy imaging revealed osteocytic cells embedded in matrix, with cellular processes, and a defined pericellular space abutting the matrix and cell bodies (Fig. 2b). SCD-O cells recreate the essential spatial context of the osteocyte’s *in vivo* environment.

The stellate morphology of osteocytes is critical to their function, enabling cell-cell connections and subsequent transmission of regulatory signals. Formation of osteocyte cell projections requires remodeling of the actin cytoskeleton to generate numerous actin rich extensions^29^. Staining of SCD-O cells with Alexa fluor 488-conjugated phalloidin revealed osteocytic cells positioned in mineralized nodules that displayed drastically different actin organization than cells residing outside of the nodules. Cells remaining in a monolayer on the culture dish had large cell bodies with actin filaments spanning the length of the cell (Fig. 2c). In contrast, osteocytes formed within mineralized nodules exhibited discrete actin formation within the cellular projections (Fig. 2d). Higher magnification images reveal osteocytic cells with condensed cell bodies connecting to one another via dendritic extensions (Fig. 2e), just as osteocytes *in vivo* connect to neighboring cells.

To confirm the morphological features of SCD-O cells, staining with E11, which is highly expressed on osteocytic dendritic projections^10^, was performed. While minimal staining was observed in cells outside of the nodules (Fig. 2f), strong immunostaining was seen in cells within mineralized nodules (Fig. 2g), confirming a location of osteocytic cells primarily within the mineralized matrix. Higher magnification images revealed extensive cellular projections, stained for E11, where cell processes radiated away from the osteocyte cell bodies, connecting to adjoining cells, reminiscent of osteocytes *in-vivo* (Fig. 2h). Taken together, TEM and immunostaining images show that cells formed within matrix nodules displayed characteristic morphological features of osteocytes.

### SCD-O cells respond to parathyroid hormone

**Figure 3:**
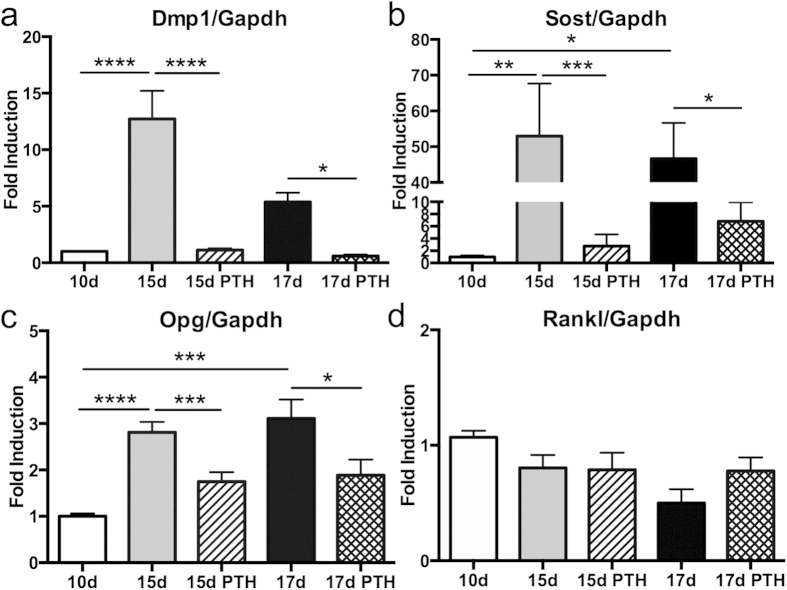
Effects of parathyroid hormone treatment on SCD-O cells. PTH treatment (1-34) of differentiated SCD-O cells (15 or 17 days) for 48 hrs decreased mRNA expression of (**a**) Dmp1, (**b**) Sost, and (**c**) Opg. PTH did not alter Rankl expression (**d**). All assays were replicated at least three times. *p < 0.05, **p < 0.01, ***p < 0.001, ****p < 0.0001

Parathyroid hormone regulates bone remodeling by directly targeting bone cells, including control of specific osteocyte secretory products^30^. SCD-O cells were treated with PTH (1-34) at either day 13 or day 15 and total RNA was harvested 48 hours later (day 15 or 17). Real-time PCR analysis showed that SCD-O cells responded to PTH with significantly decreased Dmp1 mRNA expression by 90% in 15-day and similarly by 90% in 17-day cultures (p < 0.0001 and <0.05 respectively) (Fig. 3a). Sost expression observed at days 15 and 17 (normalized to day 10), were significantly suppressed by 48 h treatment with PTH by 95% at day 15 (p < 0.001) and by 85% at day 17 (p < 0.05) (Fig. 3b). Changes in Opg were also observed where PTH significantly decreased expression by 38% at 15 days (p < 0.001) and by 40% at 17 days (p < 0.05) (Fig. 3c). Rankl expression did not change after 48 hours of PTH (Fig. 3d). These data demonstrate that SCD-O cells respond to PTH treatment in a manner consistent with *in vivo* osteocytes, a critical feature of a viable osteocyte model.

### Regulation of SCD-O cells by 1,25(OH)_2_ vitamin D_3_

**Figure 4:**
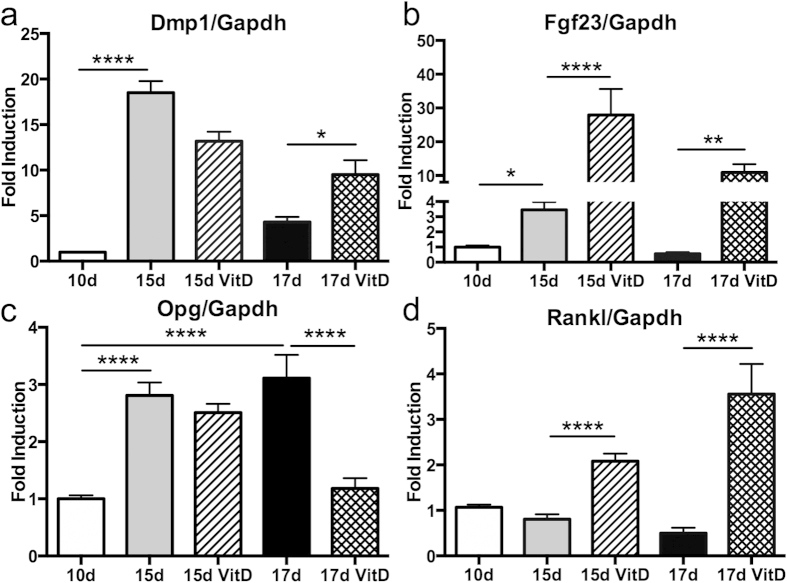
Effects of 1,25VitD_3_ treatment of SCD-O cells. 48 h application of 1,25VitD_3_ to SCD-O osteogenic cultures induced a significant increase in Dmp1 in 17 day cultures (**a**). Fgf23 (**b**) and Rankl (**c**) mRNA expression were significantly increased at both 15 and 17 days. 1,25VitD_3_ significantly reduced Opg levels in 17 day SCD-O cultures (**d**). At least three replicates were completed for each assay. *p < 0.05, **p < 0.01, ***p < 0.001, ****p < 0.0001

Osteocyte responses to 1,25(OH)_2_D_3_ orchestrate systemic changes in calcium and phosphate homeostasis by altering Fgf23 expression, a renal-targeted endocrine signal produced primarily by osteocytes^4^. At either 13 or 15 days in culture SCD-O cells were treated with 1,25(OH)_2_D_3_ for 48 hours, and total RNA was extracted 48 hours later. At day 15, 1,25(OH)_2_D_3_ treatment had a non-significant effect on Dmp1 (Fig. 4a). At 17 days, 1,25(OH)_2_D_3_ significantly increased Dmp1 expression by 29% (Fig. 4a, p < 0.05). In contrast, 48 hour treatment with 1,25(OH)_2_D_3_ significantly increased Fgf23 mRNA expression at both 15 and 17 days by 8-fold (p < 0.0001) and 20-fold (p < 0.01) respectively (Fig. 4b). Opg expression was significantly (p < 0.0001) reduced in 17 day cultures following 1,25(OH)_2_D_3_ exposure (Fig. 4c). 1,25(OH)_2_D_3_ treatment enhanced Rankl expression by 2.6-fold at 15 days (p < 0.0001) and by 7-fold at 17 days (p < 0.0001) (Fig. 4d). These data demonstrate that SCD-O cells faithfully phenocopy *in vivo* osteocyte responses to 1,25(OH)_2_D_3_.

### SCD-O cells are mechanically responsive

**Figure 5:**
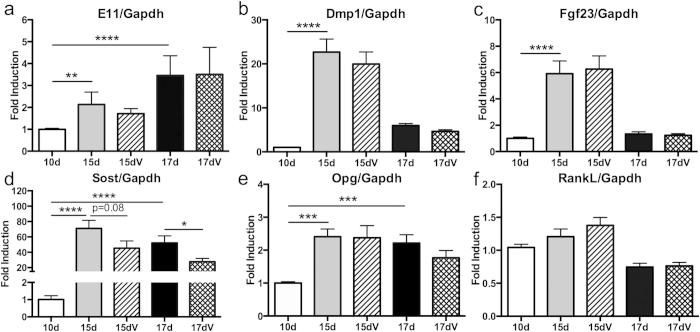
LIV reduces Sost expression in SCD-O cells. Exposure of SCD-O cells to LIV (V) for 20 min, twice a day, reduced Sost mRNA expression at both 15 (p = 0.08) and 17 days (p < 0.05) (**e**). Expression of E11 (**a**) Dmp1 (**b**) Fgf23 (**c**) Opg (**d**) and Rankl (**e**) were unaltered by LIV treatment. At least three replicates were completed for each assay. *p < 0.05, **p < 0.01, ***p < 0.001, ****p < 0.0001

As osteocytes *in vivo* are ideally positioned to sense and respond to mechanical loading^6^,^18^, we sought to determine if SCD-O cells would also respond to mechanical input. Mechanical signals have typically been delivered to *in vitro* osteocyte monolayers using fluid shear systems; however, the mechanical contribution of fluid shear delivered to the cells embedded in mineralized matrix would be non-homogenous and difficult to characterize. Instead, we applied low intensity, high frequency vibration (LIV), as the accelerations created by LIV provide a uniform and consistent mechanical stimulus over the entire culture^31^. As sclerostin expression is suppressed with mechanical loading *in vivo*^32^, we hypothesized that LIV would induce a similar response in SCD-O cells. SCD-O cells were exposed to LIV (0.7 g, 90 Hz, 20 min) twice daily (separated by 3 hours) two days prior to total RNA extraction, and on the day of harvest at either 15 or 17 days. There were no significant differences in E11 (Fig. 5a), Dmp1 (Fig. 5b), Fgf23 (Fig. 5c), Opg (Fig. 5e), or Rankl (Fig. 5f) following LIV treatment, suggesting that osteocyte differentiation and mineralization were preserved within a 3-day LIV treatment. Exposure to LIV induced a 36% reduction in Sost mRNA at 15 days in culture, demonstrating a downward trend (p = 0.08). When SCD-O cells were cultured to 17 days, a significant 47% reduction in Sost expression was observed following LIV treatment (p < 0.05) (Fig. 5d). These data support the ability of this *ex vivo* culture system to respond to mechanical signals.

### SCD-O cells secrete functional sclerostin

**Figure 6:**
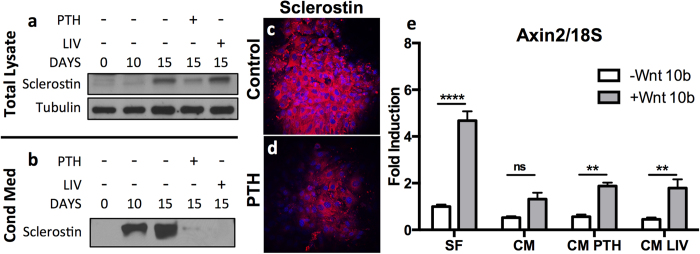
SCD-O cells secrete sclerostin. (**a**) Immunoblot of SCD-O total cell lysates revealed increased sclerostin expression at 15 days. Exposure to PTH for 48 hrs reduced sclerostin expression, whereas little to no change was seen following LIV treatment (**a**). (**b**) Sclerostin was measured in a 2 d collection of conditioned media. Treatment with PTH and LIV reduced secretion of sclerostin (**b**). Sclerostin protein production was confirmed by immunocytochemistry. Control nodules stained positively for sclerostin (**c**) staining, which decreased after 48 hr PTH exposure (**d**). (**e**) Axin2 mRNA expression from mdMSCs was determined following exposure to serum free media (SF), conditioned media (CM) from SCD-O cells collected from day 15-17 days, conditioned media from SCD-O cells treated for 48 hrs with PTH followed by 24 hrs of serum free media (CM PTH), or conditioned media from SCD-O cells treated for 4 days with LIV (2 × 20 mins daily, CM LIV). CM (50%) was added to mdMSCs overnight. The next day, mdMSCs were incubated with Wnt10b or PBS control. Addition of Wnt10b to mdMSCs incubated in SF media induced a significant increase in Axin2 expression. The elevated Axin2 response with Wnt10b exposure was ablated following overnight incubation with CM from SCD-O cells, with no significant (ns) increase over cell treated with PBS. SCD-O CM after PTH or LIV resulted in a significant increase in Wnt10b induced Axin2 expression. Blots were cropped for clarity; full-length blots are presented in supplementary figure 1. All Western blots were run under the same experimental conditions.

Sclerostin antagonizes the Wnt/LRP/β-catenin signaling axis by binding LRP5/6, blocking access to Wnt peptides^12^, resulting in suppressed osteoblast differentiation. Using Western blotting we show that sclerostin protein production was increased at day 15 compared to day 0 and day 10 (Fig. 6a). Treatment with PTH for 48 hours resulted in reduced sclerostin protein in the total cell lysate (Fig. 6a), consistent with the effect of PTH on sclerostin mRNA. In response to LIV, no discernable differences in cell lysate sclerostin were noted (Fig. 6a). To examine the ability of SCD-O cells to secrete sclerostin, conditioned media (CM) was condensed 5-fold and relative sclerostin protein production was determined by immunoblot. CM sclerostin was elevated by day 10, with greater accumulation in day 15 cultures (Fig. 6b). Treatment with PTH for 48 hours nearly ablated sclerostin secretion (Fig. 6b). Interestingly, LIV treatment also significantly suppressed sclerostin secretion (Fig. 6b), suggesting that the secretory apparatus may be mechanically regulated.

Sclerostin protein expression in response to PTH treatment was also assessed by immunocytochemistry. Staining with an anti-sclerostin antibody showed strong fluorescent signal in SCD-O cells at day 15 (Fig. 6c), whereas treatment with PTH (48 h) reduced the sclerostin-specific signal under identical conditions (Fig. 6d).

To determine if CM containing sclerostin altered Wnt/β-catenin signaling, CM was collected from serum free cultures following 48-hour exposure to PTH or after 4 days of LIV (two 20 min bouts per day), at day 17. CM from each group (no treatment, PTH, or LIV) or serum free media was added to undifferentiated mdMSC cultures. After overnight incubation with CM, mdMSCs were treated with Wnt 10b (100 ng/ml) for 3 hours before extraction of total RNA for real-time PCR analysis. Wnt 10b is a potent activator of β-catenin by its association with Lrp5/6^33^. Expression of Axin2, a down stream product of β-catenin signaling^34^, was measured to assess the level of Wnt 10b-mediated β-catenin activation. Under control conditions (serum free media), Wnt10b induced a 4-fold (p < 0.0001) increase in Axin2 expression (Fig. 6e). Overnight incubation with osteocyte CM prior to Wnt exposure dramatically attenuated the increase in Axin2 expression in response to Wnt10b treatment with no significant differences noted (Fig. 6e). CM from osteocytes treated with PTH partially, but significantly, rescued the Wnt10b Axin-2 response (3-fold increase, p < 0.01). CM from LIV-treated osteocytes also significantly rescued the Wnt 10b response with a 4-fold increase in Axin2 expression (Fig. 6e, p < 0.01). These data suggest that secreted factors from SCD-O cells effectively modulate responses to Wnt signals.

## Discussion

Osteocytes are one of the most abundant primary cell types comprising the metabolically active tissue component of the vertebrate skeleton, and are the terminally differentiated cell of the osteoblast lineage. Study of these embedded cells has proven difficult due to their structural and spatial position in bone, creating the need for *ex vivo* culture methods. The number and utility of available osteocyte cell models are limited. Current models incompletely replicate osteocyte genotype, structure, and physiology. Using isolated bone marrow MSCs, we have devised a method to generate cultures containing osteocytes that can faithfully recapitulate *in vivo* osteocyte physiology within 15 days of culture. While considerable time is required to isolate the mdMSC precursors necessary to generate SCD-O cultures, once extracted these cells can be expanded and frozen and a single vial of expanded MSCs used for up to 15 passages, enabling numerous experiments to be completed.

Osteocytes are morphologically, phenotypically, and functionally unique from their osteoblast predecessors, in part due to selective expression of genes that characterize the osteocyte phenotype. Establishing a model that expresses known osteocyte markers is key to producing a true *ex vivo* system for study of osteocyte physiology. SCD-O cells required only 15 days in osteogenic conditions to produce osteocyte-specific genetic markers including E11, Dmp1, Fgf23, and Sost. This system induces rapid and robust osteocyte gene expression in a primary culture, without complications associated with cell immortalization. Furthermore, as the mdMSCs used for this system can be made from transgenic mice^24^, SCD-O cells can be used to study the effects of specific genes on osteocyte behavior.

The SCD-O culture system is a heterogeneous model containing other MSC derived cells (e.g. MSCs & osteoblasts). The heterogeneity of this system is a limitation in that osteocyte specific effects may be difficult to distinguish from non-cell autonomous effects. However, osteocytes do not respond normally unless they are in an organ-like environment, connected to the extracellular matrix and other cell types that affect their physiology. It is for this reason that osteocyte cell lines, such as the MLO-Y4 cells, have limited utility, as environmental context affects both morphology and gene expression. Similar to our SCD-O cells, the recently described cell line IDG-SW3 also represents a non-homogenous osteogenic population^21^, but is immortalized and requires longer to attain the osteocyte phenotype. In addition to genes specific to osteocytes, expression of markers present in both osteocytes and osteoblasts were observed in SCD-O cultures. Opg and Rankl coordinately regulate osteoclast function^35^, and while Rankl is produced early in the osteogenic lineage^36^, osteocytes are thought to be the primary source of Rankl contributing to osteoclast function^2^. Our SCD-O culture system produces both of these bone-remodeling signals at early time points.

As differentiation into the osteocyte phenotype progresses, one would expect osteocyte-specific markers to continue to rise. While some osteocyte genes continued to increase, we found reductions in several genes from day 15 to 17 in culture. These included Dmp1, Fgf23, and Phex. While the reasons for the reduced mRNA levels at day 17 are unclear, each of the affected genes has a role in matrix mineralization; possibly suggesting that day 15-17 represents maximal matrix maturation resulting in a negative feedback to reduce production of factors involved in mineralization.

Interaction of osteocytes with the matrix and their spatial organization within a three dimensional environment is critical to their responses to various stimuli. TEM imaging established that SCD-O cells displayed ultrastructural characteristics of *in vivo* osteocytes: SCD-O cells were embedded in an organized matrix, had a defined lacunar space surrounding the osteocyte body, and had dendrite-like extensions. The dimensions of the osteocyte LCS are regulated by various molecules including matrix metalloproteinases (MMPs)^37^ and heparan sulfate proteoglycans^16^, which impact the response to fluid transport and mechanical loading^6^. Additionally, estrogen deficiency alters osteocyte ultrastructure^38^. SCD-O cells provide a physiologically relevant system to investigate osteocyte ultrastructure *ex vivo*, something that has not been accomplished in any other osteocyte cell model.

Isolation and identification of osteocytes were originally based on their stellate morphology. Confocal microscopy here demonstrated the presence of actin-rich cellular projections in SCD-O cells. Osteocyte actin formation was distinct from the structure of cells outside of the nodules, which remained in a monolayer. Compared to cells outside of the nodule, osteocytic cells, positively staining with E11, had more condensed cell bodies with numerous dendritic extensions that stained heavily for filamentous actin. The E11 staining was highly concentrated at the cell membrane with punctate staining of osteocytic projections seen connecting multiple cells. Cells without the nodule had little if any E11 positivity, suggesting that osteocytes only formed within the mineralized nodules.

Parathyroid hormone is a potent anabolic agent in bone. Injection of PTH (1-34) *in vivo* suppresses sclerostin expression^39^ and constitutive activation of the PTH receptor in osteocytes increases bone mass and decreases sclerostin production^30^. PTH also decreases OPG and increases RANKL in osteocytes^40^. SCD-O cells responded in similar fashion with reduction in Sost expression and secretion following PTH treatment. While Opg mRNA was suppressed, no changes were observed in Rankl expression following PTH treatment. These data contrast with PTH-mediated repression of Rankl in bone organ cultures^40^ and in stromal cells^41^. As the SCD-O cultures are hetergenous, and other cell types, including osteoblasts, produce Rankl; the response of Rankl to PTH in this system may be masked by expression from other cell types. Additionally, the Rankl response to PTH may be differentially temporally regulated in these cells compared to other models. Further studies using SCD-O cells may help elucidate the effects of PTH on Rankl across the osteogenic lineage. We also found that PTH reduced osteocyte Dmp1 expression, which plays an essential role in bone matrix mineralization. As PTH has been shown to alter bone mineral composition and collagen quality^42^, SCD-O cells may serve as an ideal model to study the effects of Dmp1-mediated regulation of matrix quality in response to PTH.

Previous studies demonstrate that 1,25(OH)_2_D_3_ induces potent increases in Fgf23 and Rankl production, while decreasing Opg and Dmp1^4^. In SCD-O cells, 1,25(OH)_2_D_3_ increased both Fgf23 and Dmp1 mRNA. The mineralized matrix surrounding SCD-O cells may influence Dmp1 production in response to 1,25(OH)_2_D_3_. Consistent with previous work, SCD-O cells displayed increased RankL with concomitantly decreased Opg in response to 1,25(OH)_2_D_3_ exposure. The osteoblast to osteocyte transition is accompanied by changes in gene expression patterns arising from qualitative and quantitative alterations in epigenetic histone marks, likely contributing to altered responsiveness to 1,25(OH)_2_D_3_^43^. SCD-O cells should be a useful tool in understanding the complex interactions of steroid hormones with the osteocyte genome, as well as providing a method to understand the alterations in FGF23 expression accompanying diseases including renal failure, vitamin D deficiency, and hypophosphatemic rickets.

Many studies have demonstrated that osteocytes are highly responsive to various types of mechanical signals including fluid shear^28^, membrane strain^44^, hypotonic swelling^18^, and vibration^17^. As SCD-O cells developed within mineralized matrix (Fig. 2), applying fluid shear would result in a non-homogenous mechanical stimulus, possibly not reaching osteocyte cell membranes within the nodules. Thus, LIV was used to mechanically stimulate SCD-O cells. We found that LIV resulted in reduced Sost expression, consistent with *in vivo* effects of mechanical load^13^,^32^.

Perhaps the most compelling contribution of the SCD-O cell model is the rapid and regulatable production of secreted sclerostin. Both PTH and mechanical load decrease sclerostin expression. Interestingly following LIV treatment, little difference was seen in sclerostin protein from total cell lysates, while sclerostin from CM was reduced to a similar level as the suppression observed following PTH treatment. Sclerostin antagonizes Wnt signaling by binding to the Lrp5 receptor on osteoprogenitors, restricting entry into the osteoblast lineage^45^. To examine if the reduced sclerostin levels in CM resulted in functional effects on Wnt/β-catenin signaling we studied its ability to prevent Wnt10b-induced Axin2 expression in undifferentiated mdMSCs. CM from osteocytes inhibited mdMSC responses to Wnt10b, while CM from both PTH and LIV treated SCD-O cells restored this response. As such, SCD-O cells serve as a powerful tool to examine both pre and posttranslational modifications of sclerostin.

In conclusion, SCD-O cells display all of the characteristics of osteocytes including the stellate morphology, lacunocanalicular ultrastructure, distinct genetic markers, and responses to hormonal and mechanical signals, all within the context of a mineralized organ-like environment. The ability to rapidly produce functional sclerostin makes this an ideal model system for elucidating mechanisms of sclerostin expression and trafficking within the cell, information that will be critical for further optimization of therapeutic treatments targeting this catabolic peptide. We also demonstrated that LIV, a clinically available treatment resulting in anabolic bone responses^46^, suppressed Sost production. This represents a novel method to offset the catabolic effects of sclerostin. Future studies using SCD-O cells and animal models may help identify new treatments, such as combination mechanical and pharmacological interventions for bone disorders. In addition to the utility of this culture system to study sclerostin, SCD-O cells may serve as an important *ex vivo* system to investigate clinical conditions affecting osteocyte secretion of FGF23, a critical regulator of renal phosphate homeostasis. The SCD-O culture system does not require immortalization, induces abundant matrix production, and rapidly attains the osteocyte phenotype. Furthermore, mdMSCs can be isolated from adult transgenic animals, enabling greater temporal control of gene silencing, overexpression, or modification, a feature no other model provides.

## Additional Information

**How to cite this article**: Thompson, W. R. *et al.* Osteocyte specific responses to soluble and mechanical stimuli in a stem cell derived culture model. *Sci. Rep.* **5**, 11049; doi: 10.1038/srep11049 (2015).

## Supplementary Material

Supplementary Information
