# Supplementary material for: Osteocyte specific responses to soluble and mechanical stimuli in a stem cell derived culture model
Source: Sci Rep. 2015 Jun 9;5:11049. doi: 10.1038/srep11049 (PMC4460727; doi:10.1038/srep11049)
Supplement: Supplementary Information [file srep11049-s1.pdf]

Osteocyte Specific Responses to Soluble and Mechanical Stimuli in a Stem Cell Derived Culture Model

William R. Thompson*[1], Gunes Uzer[2], Kaitlyn E. Brobst[2], Zhihui Xie[2], Buer Sen[2], Sherwin S. Yen[2], Maya Styner[2], Janet Rubin[2]

1. Department of Physical Therapy, School of Health and Rehabilitation Sciences, Indiana University, Indianapolis, IN 46202
2. Department of Medicine, University of North Carolina, Chapel Hill, NC 27599

Supplementary Figure 1:

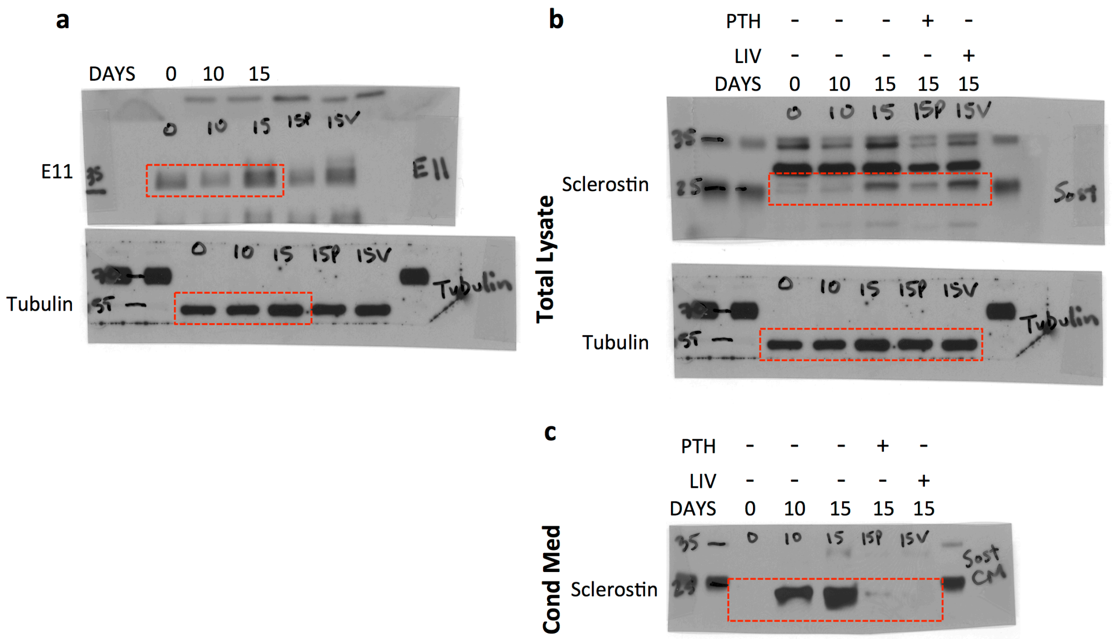

Fig. S1: Full length Western blots. (a) E11 and tubulin Westerns from Fig. 1. (b) Western blots of sclerostin and tubulin from total cell lysates from Fig. 6. (c) Western blots of sclerostin using conditioned media from Fig. 6.
